# The Effect of Sugarcane Bagasse Fiber on the Fatigue Performance of Recycled Aggregate Concrete

**DOI:** 10.3390/ma19101974

**Published:** 2026-05-10

**Authors:** Chuheng Zhong, Wenhao Deng, Jinzhi Zhou

**Affiliations:** 1 School of Civil Engineering, Architecture and Environment, Hubei University of Technology, Wuhan 430068, China; 2 Key Laboratory of Intelligent Health Perception and Ecological Restoration of River and Lake, Ministry of Education, Hubei University of Technology, Wuhan 430068, China; 3 China Railway Major Bridge Engineering Group Co., Ltd., Wuhan 430050, China; 4 Hubei University of Technology Engineering and Technology College, Wuhan 430068, China

**Keywords:** sugarcane bagasse fiber, recycled concrete, flexural strength, fatigue life, Weibull distribution, damage evolution model

## Abstract

This study tested the mechanical properties of sugarcane bagasse fiber-reinforced recycled aggregate concrete (SFRAC) with sugarcane bagasse fiber (SF) volume fractions of 0.5%, 1.5%, and 3%, and recycled coarse aggregate (RCA) replacement rates of 20%, 40%, and 60% by the mass of coarse aggregate. Evaluated parameters included compressive strength and flexural strength. Based on the mechanical performance test results, seven specimens with superior performance were selected for further flexural fatigue testing. This identified the optimal SF and RCA replacement ratios that balance mechanical performance, fatigue resistance, and economic/environmental considerations. The study concluded that sugarcane bagasse fiber significantly enhances the mechanical properties of recycled aggregate concrete (RAC). At a fiber volume concentration of 1.5%, compressive strength increased by up to 15.1%, while flexural strength improved by up to 24.6%. Regarding fatigue performance, the flexural fatigue life of SFRAC increased synchronously with rising SF content, with test results highly consistent with the three-parameter Weibull distribution. Based on this, the P-lgS-lgN equation and the S-$\lambda_{f}$-N equation incorporating failure probability and fiber parameters were derived. A fatigue strain-based damage evolution model was established to predict damage levels and remaining life of SFRAC. SEM experiments confirmed SF’s reinforcing effect on SFRAC at the microstructural level. These studies demonstrate that SFRAC with a 1.5% SF content and 40% RCA substitution offers optimal performance and environmental sustainability.

## 1. Introduction

Against the backdrop of accelerating global climate change and the rapid depletion of non-renewable resources, the construction industry, as one of the major sectors in terms of resource consumption and carbon emissions, faces an urgent need to transition toward a low-carbon, circular economy model [1]. Traditional concrete is a composite building material composed of multiple different raw materials, with coarse aggregates and fine aggregates accounting for 75% of the total weight of concrete. To meet the growing demand for aggregates, up to 50 billion tons of natural river sand and gravel are mined annually [2,3]. River siltation, mountain devastation, and ecosystem degradation are problems that result from the extraction of natural aggregates. Carbon emissions are elevated as a result of the energy-intensive mining and transportation processes, which emit substantial quantities of CO_2_. Nevertheless, the global production of construction debris is increasing annually as a result of the progress of urbanization and modernization in a variety of countries [4]. Traditional landfill disposal not only occupies land but also pollutes the soil. After being pulverized and processed, waste concrete is transformed into recycled aggregate and utilized to manufacture new concrete, which is referred to as recycled concrete [5]. This approach substantially mitigates landfill disposal and dependence on natural mineral resources, thereby safeguarding non-renewable resources.

It is widely recognized that recycled aggregate concrete (RAC) performs inferiorly in comparison to conventional natural concrete (NAC) [6]. On the one hand, the internal structure of recycled aggregates is subjected to a significant number of micro-cracks during the pulverizing process. On the other hand, the uneven surface of recycled aggregates is coated with old, hydrated cement mortar, which results in higher porosity and water absorption [7,8]. The efficacy of new concrete is adversely affected by the addition of recycled aggregates in practical applications [9].

Research has shown that the incorporation of fibers can significantly improve the mechanical properties of cement-based composite materials [10,11,12]. The mainstream types of fiber admixtures include steel fibers, synthetic fibers, and plant fibers. However, while steel fibers significantly enhance concrete’s mechanical properties [13], cracking during concrete service leads to steel fiber corrosion and subsequent strength degradation. This prompted the development of synthetic fibers with superior corrosion resistance. Yet, due to their high cost, attention has shifted toward plant fibers, which are abundant and affordable [14]. Natural plant fibers possess unique microstructures and interfacial bonding mechanisms, demonstrating superior performance compared to most industrial synthetic fibers. Examples include sisal, coconut, bamboo, and hemp fibers [15,16,17]. These fibers are low-cost, have short regeneration cycles, and are easily accessible, making them a promising option for achieving green and sustainable construction [18].

The United Nations Food and Agriculture Organization’s data indicates that the global sugarcane production in 2021 was approximately 2.1 billion tons. Approximately 3 tons of sugarcane fiber are generated for every 10 tons of sugarcane that is processed [19]. For example, in Bangladesh, sugarcane juice is a popular beverage that generates approximately 800,000 tons of sugarcane bagasse annually. This bagasse is typically processed in open-air facilities, causing environmental pollution [20]. Simultaneously, it represents a valuable resource. Reports indicate that incorporating SF into polymers can impart various mechanical properties, with current applications spanning automotive components, textiles, and construction materials. This demonstrates SF’s significant contribution to sustainable material development [2]. The application of SF in cement-based composite materials, such as mortar [21,22] and concrete [23,24], has been the subject of numerous investigations. Research suggests that the mechanical properties of cement-based materials can be improved and their porosity can be reduced by incorporating SF [25]. However, due to SF’s hydrophilic nature, its performance under load conditions is suboptimal. Therefore, alkali treatment is employed to enhance SF’s surface roughness, thereby improving the bond strength between SF and the substrate [20]. Datchossa et al. [26] discovered that the density of SF increased by 19%, and its physical aspect and primary geometric shapes changed as a result of thermal treatment and NaOH treatment. AC et al. [27] discovered that mortar composite materials can be strengthened by the addition of even tiny quantities of SF, following treatment with specific reagents. Philip et al. [28] found that SF reduces the corrosion current density in concrete by reinforcing its microstructure and enhances the mechanical strength of the concrete. Nevertheless, the porosity of the cement matrix is considerably increased by an excessive amount of SF content, resulting in a decrease in strength [29]. Consequently, it is of the utmost importance to ascertain the optimal SF content in concrete.

Concrete fatigue failure denotes the occurrence of failure in concrete materials subjected to alternating stresses far below their strength or yield limits [30]. With the widespread application of concrete, concrete structures subjected to fatigue loads are becoming increasingly common, such as bridge decks, airport runways, and highway pavements. Fatigue damage induced by vehicular loads is a primary element influencing structural safety, presenting significant difficulties to structural safety. Riyar et al. [31] and Cui et al. [32] discovered that the incorporation of polypropylene fibers into concrete markedly lowers the rate at which fatigue strain increases and substantially enhances the fatigue deformation capacity of the material. Regarding the fatigue properties of plant fiber concrete, de Andrade Silva et al. [33] investigated the tensile fatigue behavior of cement composites reinforced with long-aligned sisal fibers. They found that at 50% ultimate tensile stress, sisal fibers could prevent and bridge cracks. Liu et al. [34] discovered that grafting SiO_2_ onto straw fiber surfaces effectively enhances surface bonding strength and unit area pull-out energy, establishing a well-correlated double-logarithmic fatigue equation. Hoy et al. [35] conducted fatigue studies on hemp fiber concrete, revealing that fatigue performance increases with higher hemp fiber content. This indicates that hemp fibers effectively resist fatigue crack propagation. Huang et al. [36] demonstrated that the three-parameter Weibull distribution function serves as an effective tool for estimating the fatigue life of concrete materials in their study on the fatigue performance of agave fiber foam concrete. They also proposed an equation for predicting the fatigue life of agave fiber foam concrete under specific survival probabilities. In summary, plant fibers can effectively enhance the fatigue performance of concrete.

Although extensive research has been conducted on the effects of sugarcane waste on concrete, most studies [37,38] have focused on the impact of sugarcane bagasse ash on concrete. Nonetheless, research on the impact of SF on the mechanical characteristics and fatigue performance of recycled concrete is scarce. In fatigue testing of fiber-reinforced concrete, the enhancing effect of fibers on fatigue performance cannot be overlooked. However, most previously proposed fatigue life prediction equations for fiber-reinforced concrete fail to account for the influence of fiber parameters. We aimed to investigate the effects of SF and RCA dosages on the mechanical and fatigue properties of SFRAC, assuming that the enhancement effect of sugarcane fiber on the fatigue resistance of recycled concrete is content-dependent, and this effect can be quantitatively described through a fatigue life and damage evolution model that integrates fiber-related parameters. Compressive and flexural tests on sugarcane bagasse fiber-reinforced concrete were conducted to examine its fundamental mechanical properties. Through SEM analysis, the microstructural mechanism of SF’s reinforcement effect on RAC was revealed. Bending fatigue tests were conducted on SFRAC to analyze its fatigue life under different stress states, and a fatigue life prediction equation incorporating fiber parameters was established using a three-parameter Weibull distribution model. The effects of SF and RCA content on fatigue strain and damage evolution in SFRAC were also investigated. The aim of this study is to diminish the environmental impact of construction waste [39], encourage the recycling and repurposing of construction and agricultural waste, alleviate the greenhouse effect, and attain both economic and environmental advantages.

## 2. Material and Sample Preparation

### 2.1. Material Properties

This study employs cement and fly ash as cementitious materials. The cement is P.O. 42.5 ordinary Portland cement manufactured by Hubei Huaxin Company (Wuhan, China). The fly ash is classified as Grade I and is manufactured in Zhengzhou, Henan Province. Table 1 presents the chemical composition and physical properties of cement and fly ash. In this experiment, 0.4% (by mass of the total binder) of a high-performance water-reducing agent was incorporated. The employed water-reducing agent is a high-performance polycarboxylic acid-based formulation, exhibiting a water-reduction rate of 20%, manufactured by Shanxi Feike New Materials Co., Ltd. (Shanxi, China), to effectively diminish the water content of recycled concrete and enhance its workability. It is worth noting that while increasing the dosage of water-reducing agents (WR) can compensate for the reduced workability caused by high RCA and SF contents, it also significantly impacts the stability of the bubble spacing coefficient, thereby potentially altering the concrete strength [40]. To eliminate this confounding factor and strictly isolate the effects of RCA and SF dosages on the properties of SFRAC, the WR content was kept constant across all mixtures. Consequently, the workability of fresh concrete decreased with increasing RCA and SF content. The water utilized for mixing was tap water sourced from Wuhan.

This study utilized natural sand, natural coarse aggregate, and recycled coarse aggregate as the aggregates. Table 2 enumerates their primary performance metrics. The RCA exhibited a lower apparent density and bulk density compared to the NCA, and the water absorption rate of RCA (3.6%) was approximately four times higher than that of NCA. This is attributed to the porous nature of the old mortar attached to the RCA surface. Furthermore, the crushing index of RCA was substantially higher than that of NCA, indicating a weaker interfacial transition zone (ITZ) within the RCA. This weaker micro-structure directly results in the degradation of the mechanical properties and durability of the RAC. The fine aggregate comprised natural river sand, the natural coarse aggregate consisted of crushed limestone, and the recycled coarse aggregate was obtained from laboratory waste samples (original compressive strength of C35) through crushing, screening, washing, and drying.

**Figure 1:**
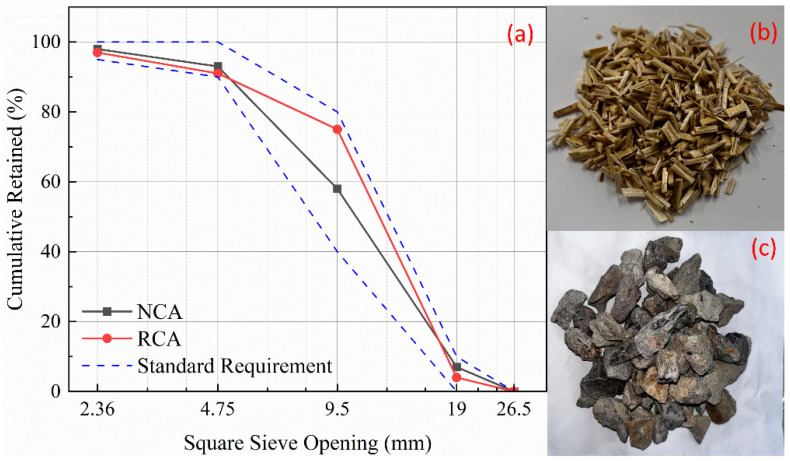
(**a**) Particle size distribution curve of aggregates; (**b**) SF; and (**c**) RCA.

The aggregates adopted continuous gradation. The particle size distribution curves of the NCA and RCA are presented in Figure 1a. The morphologies of the sugarcane bagasse fiber and RCA are shown in Figure 1b and Figure 1c, respectively.

The SF utilized in this investigation was derived from leftover sugarcane bagasse produced during the sugar manufacturing process at a facility in Guangxi. The outer fiber and pith of the sugarcane bagasse were segregated, and the outer fiber was truncated. The subsequent stage entailed rinsing the sugarcane fiber with fresh water to eliminate residual sucrose and glucose, as leftover sugar could negatively impact the efficacy of concrete [41]. To mitigate the degradative effects of alkaline cement environments on SF, treating SF with alkaline solutions removes easily degradable lignin and hemicellulose from the fibre surface while simultaneously increasing surface roughness [41]. At the same time, to reduce the alkalinity of the cementitious matrix, this experiment replaced 25% of the cementitious material with fly ash to lower the calcium hydroxide content in the matrix [42]. Therefore, to improve SF performance, the dried fibers were immersed in a 15% Ca(OH)_2_ solution for 12 h, followed by rinsing with distilled water and drying [43]. After SF processing, the measured SF length was 8–12 mm, with a diameter of 0.8–1.2 mm.

The concrete mix design is computed in accordance with the specification JCJ55-2011, with the concrete strength grade designated as C40. The water–cement ratio for each concrete mix was set at 0.4, which is indicated in the manuscript. The mass replacement ratio of recycled coarse aggregate for natural coarse aggregate is 20%, 40%, and 60%, and the volume content of SF is 0.5%, 1.5%, and 3%. NAC refers to normal concrete. Table 3 displays the mix proportions for each group, with the numeral following “R” indicating the replacement rate of recycled coarse aggregate, and the numeral following “S” denoting the content of sugarcane bagasse fiber. R20S0.5 signifies recycled coarse aggregate concrete with a 20% replacement rate and a 0.5% inclusion of sugarcane bagasse fiber.

### 2.2. Experiment Design

During the mixture preparation process, the recycled coarse aggregate, natural coarse aggregate, and natural river sand are first added into the mixer and mixed for 2 min. Subsequently, the cement and fly ash are added, and mixing is continued for another 2 min. While mixing continues, fibers are gradually added over a period of 2 min to ensure uniform distribution. Next, the pre-mixed water reducer and water are added to the mixture, followed by mixing for 3 min. After a uniform mixture is achieved, it is poured into the molds and vibrated until the concrete surface is free of bubbles. When the demolding conditions are reached, the specimens are removed from the molds and placed in a curing room for 28 days.

**Figure 2:**
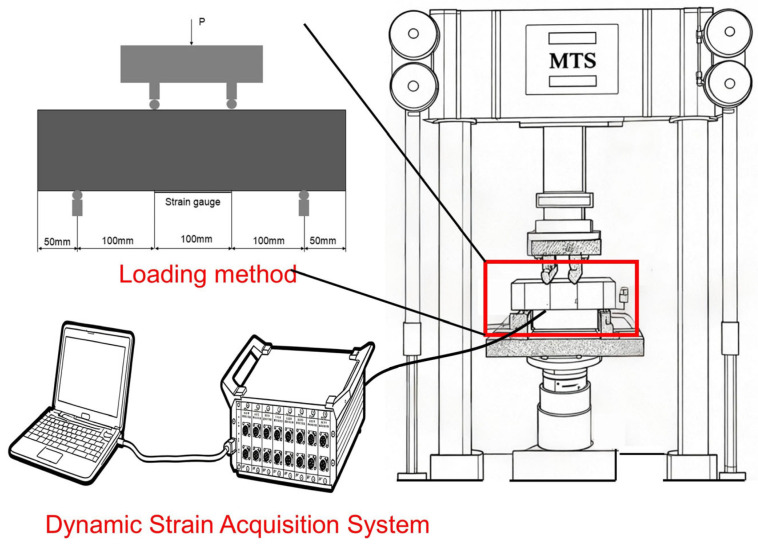
Fatigue testing methods.

Compressive strength tests were performed in accordance with the stated standards utilizing a DYE-2000S microcomputer-controlled servo pressure testing machine on specimens measuring 100 mm × 100 mm × 100 mm. The loading rate was consistently established at 0.5 kN/s. The compressive strength and ultimate load at failure were recorded. The bending flexural strength was assessed using a four-point bending test on specimens of 100 mm × 100 mm × 400 mm, utilizing a CBT1105-D type MTS microcomputer-controlled electronic pressure testing machine, in compliance with testing criteria. The loading rate was established at 0.5 kN/s. For both compressive strength tests and flexural strength tests, three specimens for each mix proportion were tested, and the average value of the test results from the three specimens was taken as the final data. If the difference between one result and the average exceeds 15%, that result is discarded and another specimen is tested. Bending fatigue tests were conducted in accordance with the Standard Test Methods for Long-Term Performance and Durability of Ordinary Concrete (GB/T 50082-2009) [44]. For each mix design, four specimens measuring 100 mm × 100 mm × 400 mm were tested on an MTS fatigue testing machine. Cyclic loading was applied using a standard sinusoidal waveform at a frequency of 10 Hz. This frequency was selected to maintain testing efficiency while reducing the heat generation associated with higher loading frequencies, thereby mitigating potential overheating effects [45], and the fatigue performance was evaluated at three stress levels: 0.6, 0.7, and 0.8 [46,47]. If the specimen remained unblemished after 2 million cycles, it was deemed to satisfy the standard criteria, and the test was concluded. The specimen was then subjected to a flexural strength test to measure its residual flexural strength. For the bending fatigue tests, to ensure the reliability of the experimental results, four specimens were tested for each concrete mix proportion at each stress level. To investigate the fatigue damage behavior of SFRAC, the bottom tensile area of the specimen was polished with sandpaper to remove surface slag and impurities, and strain gauges were attached. The DH3817F dynamic strain instrument was used to collect strain data from the specimen. This instrument has two acquisition modes: continuous and intermittent. To ensure data continuity and accuracy, the continuous acquisition mode was chosen for this experiment, as shown in Figure 2.

## 3. Results and Discussion

### 3.1. Results and Analysis of Mechanical Test

**Figure 3:**
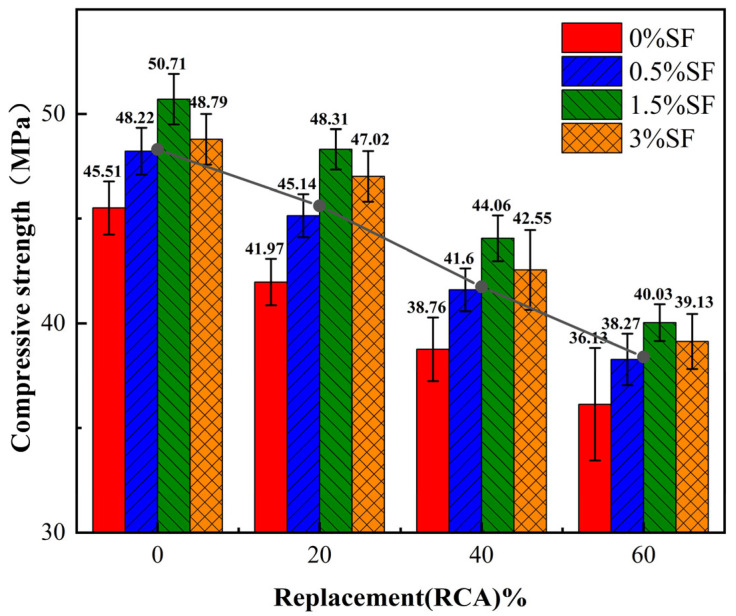
Compressive strength.

Figure 3 shows the compressive strength test results of specimens with different mix ratios. The gray lines denote overall average strength across all fiber dosages for a given RCA replacement rate. The data illustrate that the compressive strength of RAC diminishes progressively as the RCA replacement rate increases, aligning with the findings of prior studies [48]. Using specimens without SF as a reference, a 20% replacement rate of recycled coarse aggregate results in a little impact on compressive strength, exhibiting a reduction of merely 7.8%. When the replacement rate surpasses 20%, the impact on compressive strength becomes pronounced, with specimens exhibiting 40% and 60% replacement rates seeing reductions of 14.8% and 20.6%, respectively. This is primarily due to the microcracks inherent in RCA and the aged mortar on its surface, which cause the porosity within the ITZ of recycled concrete to increase as RCA is added [49], consequently leading to a decrease in strength. Conversely, the incorporation of SF reduces the porosity of the RCA matrix, resulting in a denser structure and enhanced compressive strength [25]. With a constant RCA replacement rate, the compressive strength of the specimens initially rises and thereafter declines as the SF content increases. When the SF content is 0.5%, the compressive strength of the specimens exceeds that of specimens without SF by 6% to 8.7%. When the SF content is 1.5%, the compressive strength of the specimens is 10.8–15.1% higher than that of specimens without SF. Nevertheless, as the SF content increased from 1.5% to 3%, the compressive strength diminished. The inclusion of an adequate quantity of fibers improves the interlocking force among aggregates, allowing the specimens to support loads collectively. Excessive fiber content can result in uneven fiber distribution inside the concrete, causing agglomerations that increase pore quantity and thus diminish its strength. The findings demonstrate that the integration of SF into RAC can alleviate the detrimental impacts of RCA imperfections on concrete. The ideal volume fraction of SF is 1.5%. It was also observed that the strengthening effect of sugarcane fiber appears to diminish at higher RCA replacement levels. This is primarily attributed to the “defect-compensation” principle between RCA and fibers in fiber-reinforced recycled aggregate concrete, where the strength of concrete does not exhibit a linear relationship with the RCA replacement ratio and fiber content. As the RCA replacement ratio increases, the interfacial transition zone in concrete develops a greater number and higher density of defects, which consequently requires a higher fiber content to compensate for these deficiencies [50]. This observation is consistent with the results shown in the strength prediction model for fiber-reinforced recycled aggregate concrete established by [51].

**Figure 4:**
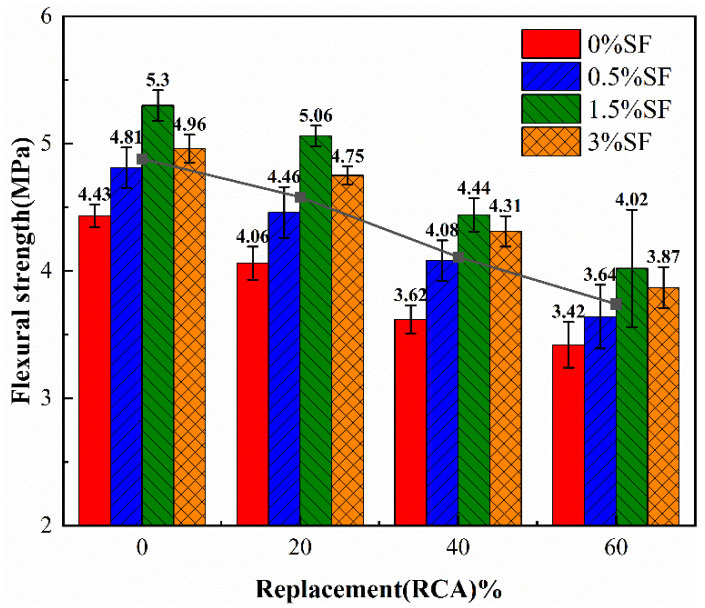
Flexural strength.

Figure 4 illustrates the flexural strengths of specimens with varying mixing ratios. The flexural strength of specimens with 0.5% SF added is 6.4–12.7% more than that of specimens without SF, whereas specimens with 1.5% SF exhibit a flexural strength increase of 17.5–24.6% compared to those without SF. This mostly occurs because, during the fracture initiation phase, when microcracks develop in concrete under bending, SF will create physical bridges on either side of the fissures, mitigating the tensile stress concentration and postponing crack propagation. In the crack extension stage, even if the crack expands, the fiber still transmits stress through plastic deformation or the pull-out process to delay the structural damage. However, when the fiber doping is increased to 3%, the decrease in flexural strength may be due to the excessive fiber content, which forms agglomerates, resulting in a reduction in strength. Based on the results of fundamental mechanical property tests, the compressive strength and flexural strength of R40S1.5 are essentially comparable to those of NAC. Research by Ohemeng et al. [52] indicates that the production cost per ton of RCA is 40% lower than that of natural coarse aggregate, with a 97% improvement in environmental benefits. In this experiment, compared to R20S1.5, R40S1.5 shows reductions in compressive strength and flexural strength of only 8.8% and 12.3%, respectively, while the RCA content is doubled. From a comprehensive analysis considering economic, environmental, and mechanical performance perspectives, R20S1.5 does not demonstrate a significant advantage. Compared to R40S0.5, R40S1.5 exhibits improved mechanical strength and a higher SF content, which helps reduce the emission of harmful substances such as carbon monoxide and nitrates during the stockpiling and incineration of bagasse waste [53]. However, the various strength indices of R60S1.5 are all lower than those of NAC, indicating a clear degradation in mechanical performance. Furthermore, SF offers significant cost advantages; in China, its raw material cost is only 25% of that of purpose-grown jute fiber. Environmentally, SF embodies a paradigm shift from “resource extraction” to “waste valorization.” As an inevitable by-product of the sugar industry, its utilization avoids the land, water, and agrochemical burdens associated with the cultivation and processing of traditional plant fibers such as sisal and jute. Therefore, given the premise of ensuring no significant degradation in fundamental mechanical properties, R40S1.5 was selected as the representative optimal mix for subsequent durability and fatigue evaluations, balancing economic and environmental benefits.

**Figure 5:**
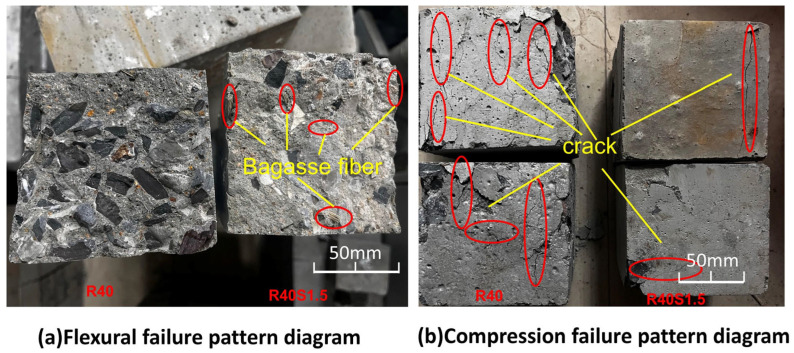
Specimen fracture surface.

During the mechanical tests, the experimental phenomena and fracture surfaces of RAC and SFRAC were compared to analyze the effect of SF on RAC. The fracture surface of the specimen is shown in Figure 5.

There are two differences between RAC and SFRAC in the process of flexural test: (1) The time from the appearance of cracks at the bottom of RAC specimens to the fracture of specimens is very short; the initial cracks will be expanded upward dramatically to form a penetrating crack, and the load readings rise rapidly, presenting obvious brittle damage. However, the time from the beginning of the crack to the fracture of the SFRAC specimen is relatively slow; the initial crack gradually extends and spreads upward, and the load reading rises steadily, showing a certain degree of ductility, which indicates that the admixture of SF improves the ability of the RAC to resist deformation and increases its flexural strength. (2) Figure 5a shows that the fracture surface of RAC is smoother and flatter than that of SFRAC. The fracture surface of RAC consists of fractured coarse aggregate and mortar, whereas that of SFRAC is concave and convex with exposed and pulled-out fibers. Additionally, SFRAC has fewer fractured coarse aggregates compared to RAC. This is because the cellulose fibers are uniformly distributed in the RAC and connected to the matrix. During crack propagation, the SFs act as bridges. They are subjected to tensile stress across the cracks, leading to deformation, pull-out, and fracture of the fibers, thereby inhibiting crack development.

Figure 5b illustrates that during the compressive strength test, the failure of RAC was accompanied by more large cracks, with the specimen breaking into fragments, resulting in a more severe failure mode and exhibiting obvious brittle failure characteristics. Conversely, the SFRAC specimens demonstrated a “cracked but not fragmented” phenomenon, preserving superior overall integrity with at most one or no discernible through cracks on the surface. This is ascribed to the elevated tensile strength of SF. When fissures develop in concrete, the SF can absorb a portion of the lateral strain, thus diminishing the rate and frequency of fracture propagation, augmenting the concrete’s ductility, and consequently enhancing its compressive strength to a certain degree.

### 3.2. Microstructure Analysis of SFRAC

**Figure 6:**
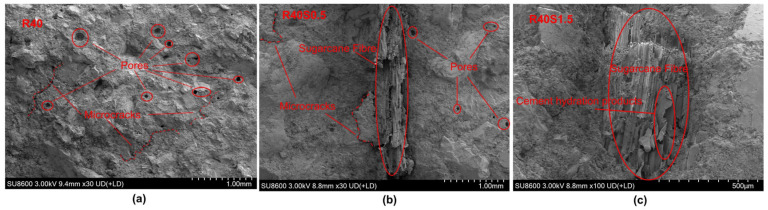
SEM images of (**a**) R40; (**b**) R40S0.5; (**c**) R40S1.5.

To investigate the microstructure and bonding properties of SFRAC, SEM analysis was performed on the fatigue fracture surfaces of R40, R40S0.5, and R40S1.5. Qualitative observations suggest that the number of pores and voids appears to decrease with increasing SF content (Figure 6a–c). This trend is consistent with the widely reported filling and bridging effects of microfibers [54].

As shown in Figure 6b,c, SFs are enveloped by hydration products, implying a certain degree of physical bonding between the fiber and the matrix. No obvious microcracks were observed at the interface, suggesting the formation of a relatively dense ITZ, which may contribute to the enhancement of flexural properties [55]. These microstructural features are visually consistent with the improved flexural properties discussed earlier.

### 3.3. Flexural Fatigue Life

#### 3.3.1. Results of Flexural Fatigue Test

The fatigue tests were conducted using specimens from the same batch as those employed in the flexural strength testing. It should be clarified that the primary purpose of the mechanical property tests was to serve as a screening process. This step was designed to exclude mixtures that failed to meet the required mechanical strength standards or deviated from the environmental objectives (for instance, R60S1.5 exhibited evident mechanical degradation). Consequently, the subsequent fatigue testing aimed to evaluate the fatigue performance of the screened mixtures that were both environmentally friendly and mechanically qualified. Based on the comprehensive analysis of mechanical and environmental performance discussed in Section 3.1, seven mixtures were selected for further fatigue performance investigation. The upper limit of the fatigue cycle load was set to S times the flexural damage load, while the lower limit was set to 0.1S times. In practical engineering, stress levels S typically do not exceed 0.5. However, using stress levels below 0.5 in experiments would consume substantial labor, time, and financial resources. Therefore, this study selected stress levels of 0.6, 0.7, and 0.8 for testing. Due to the substantially different nature of the fatigue test results, four identical specimens were fabricated for bending fatigue tests for each experimental group. Table 4 delineates the specific groupings together with the upper and lower thresholds of cyclic loading. The results of the fatigue test are presented in Table 5.

**Figure 7:**
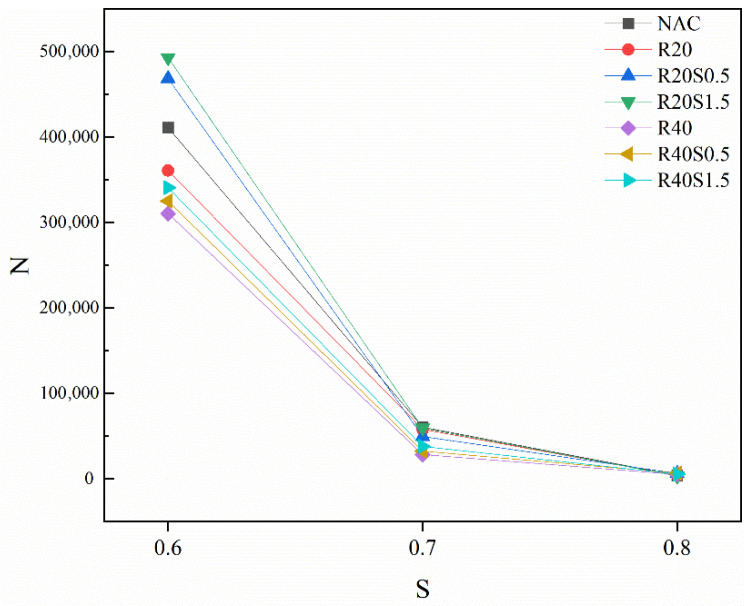
S-N curve.

Figure 7 illustrates the average fatigue life of SFRAC across various stress levels. The fatigue life of concrete diminishes as stress levels increase, as each cycle of loading induces plastic deformation and microcracking. The greater damage incurred during a single cycle at elevated stress levels hastens the accumulation of overall damage. When damage attains a critical threshold, the concrete undergoes damage. The fatigue life of R20 and R40 is significantly lower than that of NAC when subjected to the same stress level. This results from the reduced bond strength between the new paste and the existing paste of RCA. Under cyclic loading, these weak interfaces are prone to cracking and expansion, leading to a reduction in the overall stiffness of the concrete. The incorporation of SF markedly enhanced the fatigue life of R20. Adding 1.5% SFs at a stress level of 0.6 increased the average fatigue life of R20 by 36.64%. This is because the three-dimensional network structure formed by SF in concrete effectively blocks microcrack propagation paths. Through bridging action, it reduces the stress concentration factor at crack tips, thereby delaying the accumulation of fatigue damage [56]. However, no significant improvement was observed in the R40S1.5 sample, potentially related to the replacement rate of RCA. Research by Xiao et al. [57] indicates that when the RCA replacement rate is below 30%, its impact on mechanical properties is negligible. Yet, when the replacement rate reaches 30% or higher, the decline in mechanical properties becomes pronounced. At this stage, employing SF fibers with a higher volume fraction may be necessary.

In 1986, Oh, B.H. [58] conducted bending fatigue tests on ordinary concrete and found that, under a given stress level, the distribution of concrete fatigue life approximately follows the Weibull distribution. Based on the S-N equation, it was indicated that the Weibull distribution can adequately describe the fatigue behavior of concrete. Subsequent studies, such as those by Luo et al. [59], Kang et al. [46], and Huang et al. [60], have all verified this conclusion. Among them, the three-parameter Weibull distribution introduces the position parameter on the basis of the two-parameter Weibull distribution, which makes up for the limitation of the two-parameter Weibull distribution that the assumption of failure initiation point is too strong, and it is more in line with the actual fatigue action situation. Therefore, this paper adopts the three-parameter Weibull distribution to analyze the fatigue life of SFRAC. The reliability function and probability density function of the three-parameter Weibull distribution are as follows:

(1) $$P\left(N_{P}\right)=1-F\left(N_{P}\right)=\exp\left[-{\left(\frac{N_{P}-\gamma }{\eta }\right)}^{\beta }\right]$$

(2) $$f\left(N_{P}\right)=\frac{\gamma }{\eta }{\left[\frac{N-\gamma }{\eta }\right]}^{\beta -1}\exp\left[-{\left(\frac{N-\gamma }{\eta }\right)}^{\beta }\right](\gamma \leq N_{P}\leq \infty )$$

where $N_{P}$ is the specimen fatigue life; γ is the positional parameter; β is the shape parameter; and η is the dimensional parameter.

Two log-variable transformations of Equation (1) lead to the derivation of Equation (3), which is more suitable for describing the fatigue life of concrete.

(3) $$\ln\ln\left(\frac{1}{P}\right)=\beta \left[\ln\left(N_{P}-\gamma \right)-\ln\eta \right]$$

In data analysis, the median rank is used to estimate the survival probability of fatigue life. The survival probability *P* of the i-th experimental value can be expressed by Equation (4).

(4) $$P=1-\frac{i-0.3}{0.4+n}$$

In the formula, i is the serial number value of the fatigue life of a group of test pieces in ascending order; n is the total number of test pieces in a group.

Let Y = $\ln\ln\left(\frac{1}{P}\right)$, X = $\ln\left(N_{P}-\gamma \right)$, A = βlnη be simplified to Equation (5).

Y = *β*X − A (5)

The iterative method is employed to accurately calculate the distribution function and characteristic parameters of the three-parameter Weibull distribution. The location parameter γ is an unknown quantity, and from the numerical distribution, γ represents the minimum fatigue life of the specimen, i.e., when the number of fatigue cycles is less than γ, the test piece will not fail. In this study, the initial value of $\gamma_{1}$ is set to 0.5N_1_. After one fitting, $\beta_{1}$ and $\eta_{1}$ can be obtained. Simplifying Equation (1) yields the following:

(6) $$\gamma_{i}=N_{P}-\eta_{i}\exp\left(\frac{Y_{i}}{\beta_{i}}\right)$$

**Figure 8:**
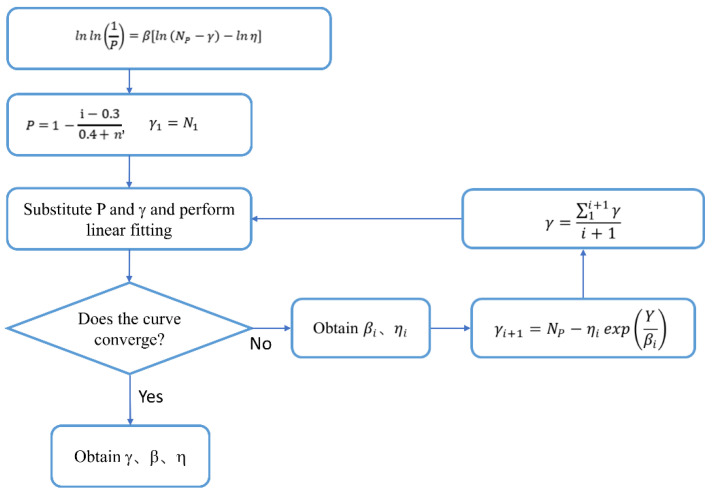
Parameter fitting calculation process.

**Figure 9:**
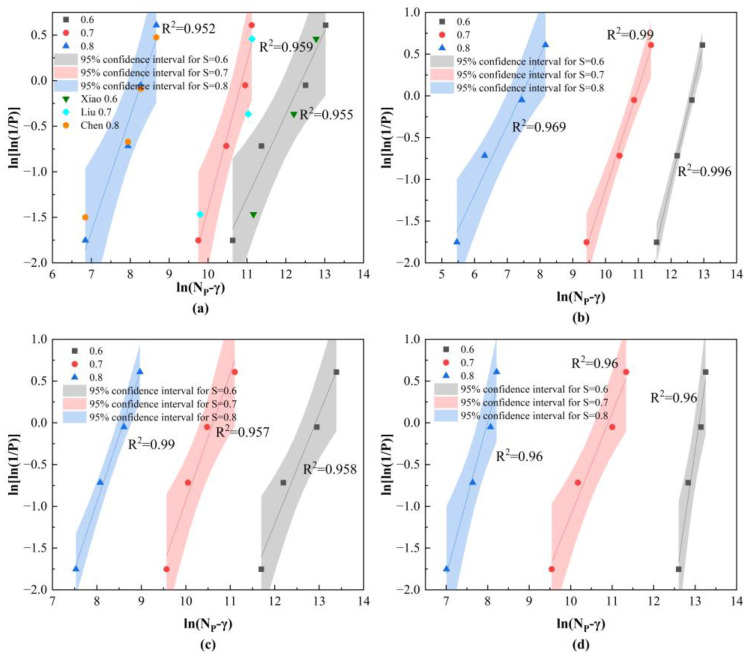
Linear regression curves of fatigue life under different stress levels: (**a**) NAC; (**b**) R20; (**c**) R20S0.5; (**d**) R20S1.5.

Each fatigue failure life corresponds to a position parameter $\gamma_{i}$. The mean value derived from each iteration serves as the updated position parameter, which is thereafter replaced as Equation (5) for iterative computation until convergence is achieved. The calculation process is illustrated in Figure 8. Table 6 presents the distribution parameters acquired at various stress levels, whereas Figure 9 illustrates the linear regression curves of fatigue life for each group of specimens across different stress levels. The correlation coefficients presented in Table 6 reveal that all values are above 0.95, indicating that the function curves illustrated in Figure 9 display strong correlation. This demonstrates that the three-parameter Weibull distribution model is applicable for characterizing the bending fatigue life of SFRAC.

To validate the distribution parameters obtained from the fitting, fatigue data from Zhong C et al. [61], Liu et al. [34], and Zhong C et al. [62] and the distribution parameters derived in this study were substituted into Equation (6) and plotted on the linear regression curve diagram. To eliminate the influence of RCA and SF, validation was performed solely on the fatigue life of NAC. As shown in Figure 9 (NAC section), the converted data points from the fatigue life obtained in the three-person experiment are uniformly distributed above and below the linear regression curve of this experiment. This demonstrates that the parameters fitted in this experiment possess high precision.

While the Weibull distribution remains a valid statistical descriptor for SFRAC, the incorporation of RCA and SF alters the underlying damage mechanisms. Research by You et al. [63] indicates that RCA introduces multiple weak interfacial transition zones (ITZs), accelerating initial damage development compared to NAC. Conversely, SF modifies crack propagation through bridging effects [64]. Crucially, the dosage sensitivity must be considered. Studies by Tan et al. [64] and Lifshitz and Ribakov [65] demonstrate that exceeding an optimal fiber volume fraction can lead to fiber clustering and matrix saturation, potentially negating reinforcement benefits. Therefore, while the NAC validation confirms the model’s fundamental structure, the parameters for SFRAC must be recalibrated to reflect these specific material interactions.

#### 3.3.2. *S*-N-*P* Equation of Flexural Fatigue

In the examination of material fatigue performance, varying stress levels, S, are applied, and the associated average fatigue life, N, is recorded, thereby establishing a quantitative relationship between the two. In practical engineering applications, the number of cyclic loads that components must withstand continues to increase, necessitating the evaluation of fatigue performance under lower stress levels. However, as acknowledged in this study, direct experimental verification at these low stress levels (<0.5) is often impractical due to the excessively long testing durations involved. Consequently, rather than relying on deterministic extrapolation, it is imperative to adopt reliability theory and formulate a P-lg*S*-lgN equation that incorporates the survival rate. This probabilistic approach serves as a scientific bridge to estimate the failure risk under service conditions. Moreover, in the analysis of concrete materials, the formulation of such fatigue equations must strictly account for physical boundary conditions to ensure the model remains robust when extrapolated beyond the tested high-stress range. The parameters governing the conditions for concrete are as follows:

(7) $$\left\{\begin{array}{c} N=1,S=1\\ N\to \infty ,s\to 0 \end{array}\right.$$

The double logarithmic fatigue equation is as follows:

(8) lgS=lgA−BlgN

where A and B are regression coefficients. When the stress levels are 0.6, 0.7, and 0.8, the corresponding logarithms of the stress levels are −0.2218, −0.1549, and −0.0969.

**Figure 10:**
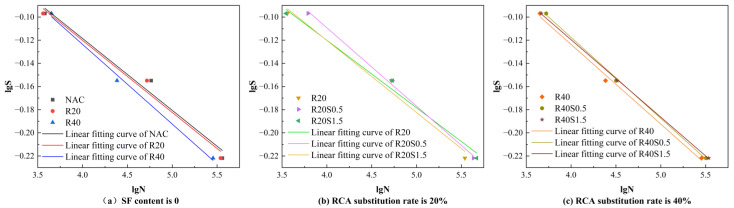
*S*-N curve with a survival probability of 0.5, S = 0.6.

The bending fatigue life of concrete at any survival rate, *P*, can be derived from Equation (9). Utilizing Equation (8) to correlate the fatigue life, N, and stress level, S, for varying survival probabilities yields the fatigue equations for SFRAC with distinct fiber content at a survival probability of 0.5, as presented in Table 7, alongside the *S*-N-*P* curves for SFRAC with different fiber content at the same survival probability, illustrated in Figure 10.

(9) $$N=\gamma +\eta {\left(-\ln P\right)}^{\frac{1}{\beta }}$$

Figure 10a indicates that at *P* = 0.5 and S = 0.6, the fatigue life decreases by 5.3% and 21.2% respectively when the RCA substitution rate is 20% and 40% compared to NAC. The primary reason is likely that the inherent microcracks in RCA accelerate the crack propagation stage, and these microcracks increase with the substitution rate, leading to a further reduction in fatigue life. Figure 10b,c demonstrate that fatigue life increases with rising fiber content. This is primarily attributed to the bridging effect of fibers: as fiber content increases, crack propagation is restricted, consequently enhancing fatigue life.

Bending fatigue strength is a critical design criterion; performing an infinite number of cyclic tests on materials experimentally is impractical. Researchers commonly define the maximum stress a material can endure under 2 million cyclic loads as fatigue strength, which can alternatively be represented as a percentage of the static load strength [66]. Table 7 illustrates that the fatigue strength of the specimens diminishes progressively with an increase in the RCA replacement rate, with R20 and R40 exhibiting losses of 10% and 28.57%, respectively. The incorporation of SF alleviates the detrimental impact of RCA on the fatigue strength of concrete, resulting in a 27.6% increase in the fatigue strength of R20S1.5 compared to R20. Notably, the fatigue strength of R40S1.5 remains highly stable across different failure probabilities, with values closely approaching those of NAC. This demonstrates that the incorporation of 1.5% SF effectively restores the fatigue resistance of RAC to a level comparable to conventional concrete. When this mechanical equivalence is combined with the significant economic savings (25% fiber cost reduction) and the environmental benefits of waste valorization, R40S1.5 stands out as the optimal compromise. Consequently, despite the observed fluctuations in fatigue life cycles, R40S1.5 is confirmed as the mix that best reconciles mechanical robustness, long-term durability, and sustainability.

Analyses demonstrate that the judicious incorporation of SF can improve the fatigue performance of RAC, which is considerably affected by SF. Consequently, it is essential to evaluate the impact of the SF characteristic parameter $\lambda_{f}$ on RAC [47]. The correlation among stress level, fatigue life, and fiber characteristics is articulated by Equation (10):

(10) $$s=a+b\lambda_{f}+c\lambda_{f}^{2}+d\mathrm{lg}N$$

where $\lambda_{f}=v_{f}\frac{l_{f}}{{\mathrm{d}}_{f}}$, $v_{f}$ is the fiber volume fraction, $l_{f}$ is the fiber length, and ${\mathrm{d}}_{f}$ is the fiber diameter. Using Equation (10) for multiple regression analysis of the experimental data yields Table 8.

Table 8 illustrates that, across varying survival probabilities, *S*, $\lambda_{f}$, and N demonstrate nonlinear correlations, with R^2^ exceeding 0.971, thereby confirming that the fatigue equation formulated at this juncture effectively characterizes the fatigue life of SFRAC under designated stress levels and failure probabilities. In order to further optimize the equation to make it applicable to C40 fiber-reinforced recycled concrete fatigue equations under different failure probabilities, the above parameters are converted into a single logarithmic equation related to *P*. The results are shown in Equation (11).

**Figure 11:**
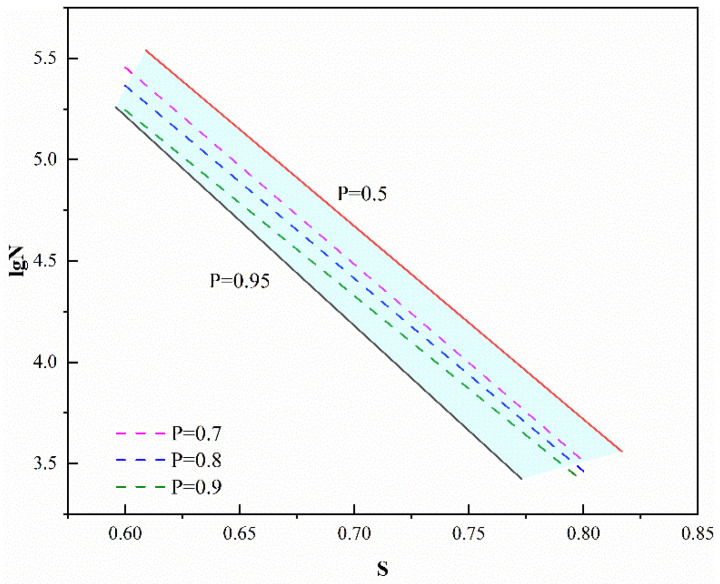
Model comparison.

Setting $\lambda_{f}$ to 0 in Equation (11), the *S*-N-*P* relationship of RAC can be obtained. As shown in Figure 11, the *S*-lgN equations calculated by the model within the *P* range of 0.5 to 0.95 can encompass the conventional *S*-lgN equations at *P* values of 0.7, 0.8, and 0.9. The model has a wide coverage and higher practicality, and the consideration of fiber parameters in the model facilitates the application of SFRAC.

(11) $$S=(-0.1575P+1.25325)+(0.3525P-0.00442)\lambda_{f}+(-2.6775P+0.31758)\lambda_{f}^{2}+(0.01525P-0.11104)lgN$$

### 3.4. Damage Evolution of Flexural Fatigue

Damage constitutes an irreversible phenomenon within a substance. The fatigue strain of concrete indicates its fatigue damage under cyclic loading. The deformation of concrete under cyclic fatigue loading is generally depicted by a strain–cycle ratio curve. The cycle ratio n represents the proportion of cycles at a specific moment in the fatigue process relative to the total number of cycles. $\varepsilon_{max}$ represents the fatigue strain at the greatest load of each cycle, whereas $\varepsilon_{min}$ denotes the fatigue strain at the minimum load of the cycle.

#### 3.4.1. Flexural Fatigue Strain Analysis

**Figure 12:**
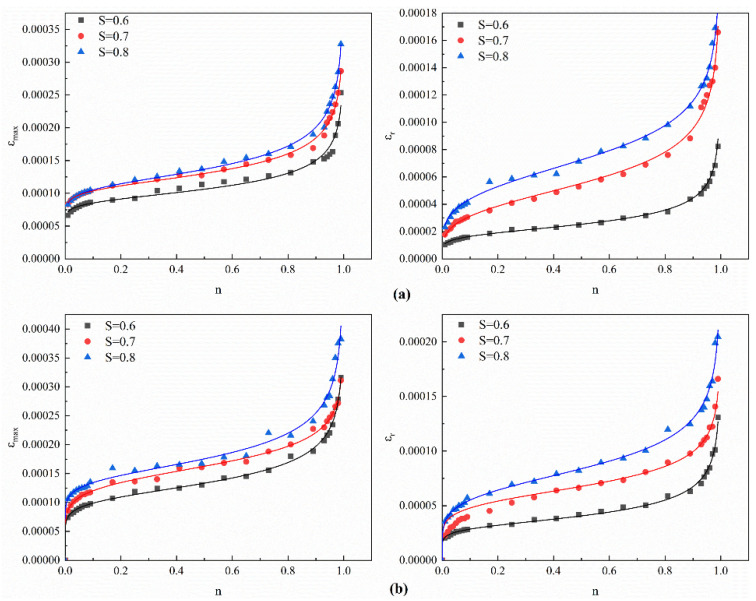
ε-*n* curve: (**a**) R20; (**b**) R20S1.5.

Given that the minimum load in this study is merely one-tenth of the maximum load, nearing a zero-load condition, $\varepsilon_{min}$ is approximately equivalent to the residual strain $\varepsilon_{r}$ of each loading cycle. Consequently, $\varepsilon_{min}$ is regarded as the residual strain $\varepsilon_{r}$ for each loading cycle in this research. Note that the strain and damage analysis herein focuses on the R20 series and R40 group. As R40S1.5 was previously validated as the optimal mix via fatigue strength, this section aims to elucidate the distinct roles of RCA and SF by isolating their effects, clarifying the underlying mechanisms without redundancy. The $\varepsilon_{max}$-*n* curves and $\varepsilon_{r}$-*n* curves derived from the four experimental sets at varying stress levels, along with their fitted values, are presented in Figure 12.

As illustrated in Figure 12, the fatigue damage process of concrete can be categorized into three primary stages: The initial stage involves the onset of microcracks, during which the strain value progressively escalates with minor increases in the cycle ratio. This stage signifies the emergence of microcracks within the specimen due to cyclic loading, which induces stress concentration and facilitates the propagation of microcracks, thereby augmenting their quantity. The subsequent stage is characterized by crack propagation, wherein strain varies at a constant rate, indicating that microcracks progressively evolve into macroscopic cracks under cyclic loading. In this phase, the specimen absorbs substantial energy, with a prolonged duration and a gradual increase in strain; the subsequent phase is marked by a swift escalation in fatigue strain, where strain escalates sharply until concrete failure transpires, characterized by the interconnection and expansion of microcracks within the specimen, ultimately resulting in failure and fracture. The analysis of the maximum fatigue strain curve relative to the cycle ratio revealed that the incorporation of SF significantly enhanced the fatigue characteristics of recycled concrete, chiefly attributable to the fibers’ bridging effect. With an increase in fiber concentration, crack propagation is inhibited, resulting in a diminished strain rate. At stress levels of 0.6, 0.7, and 0.8, the maximum fatigue strain of R20S1.5 rose by 24.9%, 8.7%, and 16.8%, respectively, in comparison to R20. Moreover, as the stress level, S, escalated, the fatigue strain of all test specimens augmented, and the rate of strain growth progressively accelerated. Under elevated stress levels, the velocity of crack propagation inside the specimens increased. Compared to R20, R20S1.5 exhibits superior ductility, with a larger proportion of the second stage during fatigue and a more gradual strain increase, demonstrating that the addition of SF prevents sudden failure of the concrete.

Scholars, both domestic and international, have extensively researched the development patterns of fatigue strain–cycle ratio curves for concrete and have created various valid fatigue strain equations [67]. This research examines fatigue strain data and uses Equation (12) for fitting and analysis of the fatigue strain:

(12) $$\varepsilon =en^{f}\times {\left(1-n\right)}^{g}$$

where *n* is the cycle ratio of the test specimen; *e*, *f*, and *g* are coefficients; and ε is the fatigue strain.

The fitting results are presented in Figure 12. The coefficients *e*, *f*, and *g* of the fitting equations for fatigue strain at various stress levels, as well as the correlation coefficients, R^2^, are presented in Table 9. The fitting efficacy of the equations for each group is commendable, with correlation coefficients R^2^ above 0.95. The fitting effects of the specimens within each group are comparable, suggesting that the formulated equations accurately represent the correlation between fatigue strain and cycle ratio in sugarcane bagasse fiber-reinforced concrete.

#### 3.4.2. Damage Evolution Equation

Damage within fiber-reinforced concrete refers to the entire process of micro-cracks forming within the concrete material and gradually developing into cracks, leading to failure. This process is characterized by the continuous accumulation of residual strain within the concrete. The cumulative residual strain within the concrete can effectively represent the damage process occurring within the concrete material.

Define the damage variable based on the definition of residual strain due to fatigue, as shown in Equation (13):

(13) $$D=\left\{\begin{array}{c} 0,\varepsilon_{n}^{r}\leq \varepsilon_{D}^{r}\\ \frac{\varepsilon_{n}^{r}-\varepsilon_{D}^{r}}{\varepsilon_{f}^{r}-\varepsilon_{D}^{r}},\varepsilon_{D}^{r}\leq \varepsilon_{n}^{r}\leq \varepsilon_{f}^{r} \end{array}\right.$$

**Figure 13:**
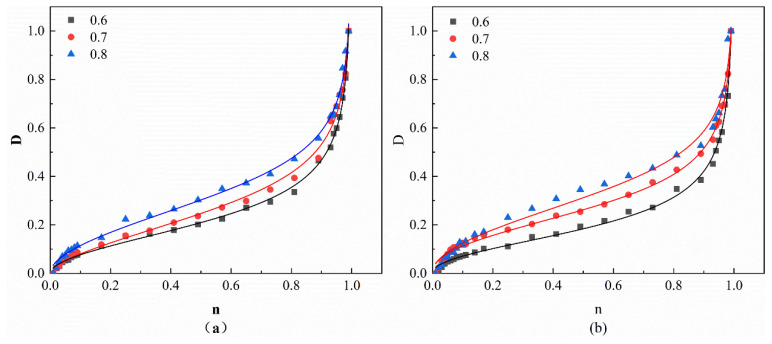
Fatigue damage variables and fitting curves: (**a**) R20; (**b**) R20S1.5.

Among these, $\varepsilon_{n}^{r}$ is the instantaneous residual strain, $\varepsilon_{D}^{r}$ is the residual strain at the onset of damage in the specimen, where $\varepsilon_{D}^{r}$ is taken as the fatigue residual strain value when *n* = 0.01 in this study, and $\varepsilon_{f}^{r}$ is the cumulative residual strain at the final failure of the specimen. When $\varepsilon_{n}^{r}$ < $\varepsilon_{D}^{r}$, macro-scale damage evolution has not yet begun; when damage begins, $\varepsilon_{n}^{r}$ = $\varepsilon_{D}^{r}$, at which point D is 0; as damage develops, $\varepsilon_{n}^{r}$ > $\varepsilon_{D}^{r}$, until $\varepsilon_{n}^{r}$ = $\varepsilon_{f}^{r}$, at which point the specimen fails and fractures. According to existing research [67], this injury definition has been validated to meet the fundamental requirements of generalized injury variables and has been widely adopted. Using Equation (13), the damage variable results for each group of specimens are calculated, as shown in Figure 13.

In addition, the damage variable of the test piece can also be obtained through Equation (14) [59].

(14) $$D=\frac{\mathrm{e}{\left(\frac{N}{N_{f}}\right)}^{f}\cdot {\left(1-\frac{N}{N_{f}}\right)}^{g}}{\varepsilon_{f}^{r}-\varepsilon_{D}^{r}}=e^{\prime }{\left(\frac{N}{N_{f}}\right)}^{f^{\prime }}\cdot {\left(1-\frac{N}{N_{f}}\right)}^{g^{\prime }}$$

Among these, *e*′, *f*′, and *g*′ are fitting parameters, *N* is the current number of cycles, and *N_f_* is the fatigue life. The experimental data were fitted using Equation (14), and the fitting results are shown in Figure 13 and Table 10.

As can be seen from Figure 13, the damage variable curve exhibits a three-stage cumulative process with continuous growth. In the first stage, the damage variable increases rapidly and then gradually decreases. In the second stage, the growth rate slows down, exhibiting a linear damage evolution process, which is consistent with the conclusions of strain analysis. This is primarily because, during the second stage, the specimen absorbs energy and gradually expands microcracks, a process that takes a long time and results in slow damage growth. In the third stage, damage suddenly accelerates, leading to specimen failure. This demonstrates that damage in concrete accumulates continuously under cyclic loading until it reaches a critical value, at which point the concrete loses its load-bearing capacity. In Equation (14), the parameter *e*′ determines the cumulative rate of fatigue damage, representing the slope of the damage curve in the second stage. At a stress level of 0.6, the values of *e*′ for R20, R20S0.5, and R20S1.5 gradually decrease, indicating that the addition of SF in RAC can effectively reduce the development rate of fatigue damage in the second stage through a bridging effect. However, the *e*′ value of R40 is 11.8% higher than that of R20. This is primarily due to the inherent defects in recycled aggregates, which have more initial voids and microcracks. The higher content of RCA accelerates the development rate of microcracks and the accumulation of damage within the specimens.

Based on the aforementioned fundamental performance tests and fatigue tests, it has been demonstrated that SF can effectively enhance the strength and performance of recycled concrete. Economically, as a by-product of the sugar industry, its raw material cost is significantly lower than that of fibers such as flax or sisal, which require cultivation, harvesting, and specialized processing, and is also lower than industrially produced synthetic fibers. For example, in China, the raw material cost per kilogram of SF is typically only 25% of that of specifically cultivated jute fiber. In terms of environmental performance, the core advantage of SF lies in its fundamental paradigm shift from “resource extraction” to “waste valorization.” Compared to traditional plant fibers like sisal or jute, which require dedicated planting, harvesting, and processing, SF is an inevitable agricultural by-product of the sugar industry. Its utilization does not occupy additional land or water resources, nor does it require the application of fertilizers or pesticides, thereby avoiding the direct environmental burden associated with obtaining fiber raw materials. This “turning waste into treasure” model not only provides the concrete industry with a low-carbon, renewable reinforcement material but also transforms the waste disposal problem of the sugar industry into a resource gain for construction materials. This achieves a reduction in environmental burden at both industrial ends, embodying the principles of a circular economy. Therefore, its environmental advantage is not merely the common attributes of being “biodegradable” or “bio-based,” but rather stems from the fact that its lifecycle begins with the valorization of waste, endowing it with an inherently low carbon footprint and zero raw material extraction cost.

Like most studies, the current research design also has limitations. Due to equipment conditions and funding constraints, the fatigue experiments in this study used a limited number of specimens, which may result in insufficient accuracy of the *S*-N curve and make it difficult to clearly show the transitional characteristics in the three-stage development of fatigue damage. However, this paper mainly investigates the effects of SF and RCA on the fatigue performance of concrete, and the influence of SF and RCA content on concrete fatigue performance can still be inferred from the trends of different concrete S-N curves and stress cycle ratio curves. In future studies, increasing the number of specimens or establishing a statistically based sample size determination method could obtain more accurate fatigue damage curves and S-N curves of SFRAC. Although the constant WR dosage ensured the stability of the air-void structure, it inevitably led to a reduction in slump and potentially affected the homogeneity of fiber distribution, particularly in the R40 and R60 series. This means that part of the observed reduction in strength and fatigue life in high-replacement groups could be attributed to the reduced workability rather than solely to the material composition. Therefore, the proposed mix proportions are most suitable for engineering scenarios with low fluidity requirements (e.g., precast elements). For applications requiring pumping or high flowability, subsequent studies should focus on optimizing the WR type and dosage to balance workability and mechanical performance.

## 4. Conclusions

Through cube compressive strength, flexural strength, and flexural fatigue tests conducted on sugarcane bagasse fiber-reinforced recycled coarse aggregate concrete, and by analyzing the experimental results, specimen damage morphology, and fatigue life damage, the following conclusions were drawn:

(1) In RAC, as the RCA replacement rate increases, the compressive strength of RAC gradually decreases. The incorporation of SF enhances the compressive strength of RAC. The compressive strength of SFRAC initially increases with the volume content of SF, subsequently declining, while the flexural strength exhibits the same pattern. The ideal replacement rate of RCA for SFRAC, to achieve a balance of strength and cost-effectiveness, is 40%, accompanied by an SF volume content of 1.5%.

(2) The three-parameter Weibull model is sufficient to characterize the bending fatigue life distributions of NAC, RAC, and SFRAC across varying stress levels and demonstrates a strong association. A cohesive single-log fatigue equation that integrates failure probability and fiber parameters was developed and juxtaposed with the double-log fatigue equation, demonstrating that the single-log fatigue equation, incorporating failure probability and fiber parameters, is more applicable and better explains the relationship between stress level, fatigue life, and failure probability.

(3) SF can increase the maximum and residual fatigue strain of RAC. The fatigue strain–cycle ratio curve of SFRAC clearly exhibits a three-stage trend; the fatigue failure process undergoes three stages: internal damage generation, internal damage stable expansion, and damage instability development. In comparison to R20, R20S1.5 demonstrates superior ductility, characterized by a greater proportion of the second stage during fatigue and a more gradual increase in strain, demonstrating that the addition of SF prevents sudden failure of the concrete.

(4) The fatigue strain equations derived from the SFRAC tests exhibit correlation coefficients over 0.95. Furthermore, by characterizing the damage variable with residual strain, the damage evolution curve for SFRAC was established, and the fatigue damage evolution equation for SFRAC was formulated through fitting. The fitting findings are rather favorable, and based on the fitting coefficients, it was inferred that the incorporation of SF can significantly diminish the progression rate of fatigue damage in the second stage.

## 5. Future Research Directions

Building upon the limitations identified in this study, the following directions are proposed for future research:

(1) Enhancing Statistical Reliability: Given the limited specimen quantity constrained by equipment and funding, future studies should employ statistically based sample size determination methods. Increasing the number of replicates will refine the accuracy of the S-N curves and capture the transitional characteristics of the three-stage fatigue damage development more clearly.

(2) Optimizing Rheology and Fiber Dispersion: This study maintained a constant WR dosage to control air-void structure, which inevitably compromised workability in high RCA/SF groups. Future work should focus on optimizing WR types and dosages specifically for high-replacement mixes to improve slump and ensure homogeneous fiber distribution, thereby isolating the true material effects from workability side-effects.

(3) Full-Scale Model Validation: The current model validation was limited. Subsequent research should extend the validation of the Weibull-based damage model across a broader range of SFRAC mixtures with varying RCA sources and fiber types to confirm its universal applicability.

## Figures and Tables

**Table 1:** Chemical composition and physical properties of cementitious materials (obtained from suppliers).

Composition	Cement	Fly Ash
	(%)	(%)
SiO_2_	19.95	47.7
Al_2_O_3_	4.79	37.53
Fe_2_O_3_	3.16	4.55
CaO	63.26	3.7
MgO	2.03	0.94
Na_2_O	0.3	0.6
K_2_O	0.39	1.62
SO_3_	2.69	0.91
P_2_O_5_	0.04	0.36
TiO_2_	3.39	1.4
Fineness (%)	0.32	10.6
Specific surface area (m^2^/kg)	380	217
Heat loss (%)	3.13	4.32

**Table 2:** Performance indicators of aggregates (obtained from suppliers).

Aggregate Type	Dimensions	Apparent Density	Bulk Density	Crushing Index	Water Absorption Rate	Silt Content
	(mm)	(kg/m^3^)	(kg/m^3^)	(%)	(%)	(%)
natural coarse aggregate	5~20	2695	1340	3.92	0.92	0.43
recycled coarse aggregate	5~20	2678	1250	10.95	3.6	0.41
natural fine aggregate	0.16~4.75	2536	1369	12.67	2.23	0.9

**Table 3:** Mix proportions of different mixtures.

NO.	Fly Ash	OPC	Water-Reducing Agent	Fine Aggregate	NCA	RCA	Water	SF
	(kg/m^3^)	(kg/m^3^)	(kg/m^3^)	(kg/m^3^)	(kg/m^3^)	(kg/m^3^)	(kg/m^3^)	(Vt, %)
NAC	105.5	316.4	1.7	660.4	1149.0	0.0	168.8	0
R20	105.5	316.4	1.7	660.4	919.2	229.8	168.8	0
R40	105.5	316.4	1.7	660.4	689.4	459.6	168.8	0
R60	105.5	316.4	1.7	660.4	459.6	689.4	168.8	0
R20S0.5	105.5	316.4	1.7	660.4	919.2	229.8	168.8	0.5
R40S0.5	105.5	316.4	1.7	660.4	689.4	459.6	168.8	0.5
R60S0.5	105.5	316.4	1.7	660.4	459.6	689.4	168.8	0.5
R20S1.5	105.5	316.4	1.7	660.4	919.2	229.8	168.8	1.5
R40S1.5	105.5	316.4	1.7	660.4	689.4	459.6	168.8	1.5
R60S1.5	105.5	316.4	1.7	660.4	459.6	689.4	168.8	1.5
R20S3	105.5	316.4	1.7	660.4	919.2	229.8	168.8	3
R40S3	105.5	316.4	1.7	660.4	689.4	459.6	168.8	3
R60S3	105.5	316.4	1.7	660.4	459.6	689.4	168.8	3

**Table 4:** Grouping conditions and upper and lower cyclic load limits.

NO.	0.6		0.7		0.8	
	f_max_ (kN)	f_min_ (kN)	f_max_ (kN)	f_min_ (kN)	f_max_ (kN)	f_min_ (kN)
NAC	8.86	0.89	10.34	1.03	11.82	1.18
R20	8.12	0.81	9.47	0.95	10.82	1.08
R20S0.5	8.92	0.89	10.41	1.04	11.90	1.19
R20S1.5	10.12	1.01	11.81	1.18	13.50	1.35
R40	7.23	0.72	8.44	0.84	9.64	0.96
R40S0.5	8.16	0.82	9.52	0.95	10.88	1.09
R40S1.5	8.88	0.89	10.36	1.04	11.84	1.18

**Table 5:** Fatigue life.

NO.	S	N_1_	N_2_	N_3_	N_4_	$\bar{N}$
NAC	0.6	239,855	285,219	468,532	650,902	411,127
	0.7	33,402	51,479	73,461	83,978	60,580
	0.8	1698	3574	4655	6589	4129
R20	0.6	208,013	299,329	410,261	526,737	361,085
	0.7	23,642	45,107	63,854	99,621	58,056
	0.8	2789	3104	4263	6076	4058
R20S0.5	0.6	241,936	319,363	540,962	773,047	468,827
	0.7	29,071	37,914	50,381	81,206	49,643
	0.8	3727	5082	7343	9716	6467
R20S1.5	0.6	328,426	405,283	539,081	600,726	468,379
	0.7	27,538	39,863	73,660	97,531	59,648
	0.8	2176	3146	4271	4771	3591
R40	0.6	152,937	258,758	370,854	540,019	330,642
	0.7	10,372	16,842	33,945	53,917	28,769
	0.8	1736	3562	6025	9809	5283
R40S0.5	0.6	213,611	285,036	379,603	423,866	325,529
	0.7	21,024	26,347	37,571	46,110	32,763
	0.8	2711	3584	7560	12981	6709
R40S1.5	0.6	200,156	268,153	329,172	566,035	340,879
	0.7	11,876	26,753	39,638	73,649	37,979
	0.8	1548	3629	6458	11145	5695

**Table 6:** Distribution parameters under different stress levels.

NO.	S	*γ*	*β*	*η*	R^2^
NAC	0.6	198,367.6267	0.91731	243,890.8654	0.9547
	0.7	16,228.62321	1.61592	52,607.09198	0.95923
	0.8	757.8592274	1.26668	4061.07099	0.95234
R20	0.6	103,457.7956	1.66066	302,782.8799	0.99598
	0.7	11,313.47864	1.20171	55,491.86127	0.98997
	0.8	2553.70768	0.83226	1668.333361	0.96909
R20S0.5	0.6	121,628.1702	1.31612	408,181.754	0.9583
	0.7	14,795.45758	1.52044	40,688.62823	0.95658
	0.8	1866.099906	1.59702	5401.534676	0.98995
R20S1.5	0.6	30,842.5368	3.37043	488,402.6237	0.96032
	0.7	13,568.57864	1.22783	54,448.09468	0.96384
	0.8	1068.756358	1.83393	2965.232764	0.95851
R40	0.6	123,367.6714	0.86971	246,888.1357	0.96421
	0.7	5204.568704	1.01077	27,328.27493	0.98177
	0.8	809.3175363	1.03028	5259.42872	0.99579
R40S0.5	0.6	105,939.165	2.05252	255,431.4543	0.96908
	0.7	10,500.00724	1.83034	25,996.09087	0.96951
	0.8	2038.201	0.79309	5142.385158	0.95716
R40S1.5	0.6	153,754.574	1.10038	221,995.18	0.97715
	0.7	5491.964246	1.00702	38,101.65312	0.98869
	0.8	664.9223982	0.9476	5878.225037	0.99432

**Table 7:** *S*-N-*P* equation with a survival probability of 0.5.

NO.	*P*	*S*-N-*P*	R^2^	Fatigue Strength (MPa)
NAC	0.5	lg*S* = 0.12957–0.06203 lgN	0.95	2.43
R20	0.5	lg*S* = 0.12882–0.06225 lgN	0.96	2.21
R20S0.5	0.5	lg*S* = 0.16045–0.06776 lgN	1.00	2.41
R20S1.5	0.5	lg*S* = 0.11313–0.0583 lgN	0.98	2.82
R40	0.5	lg*S* = 0.15155–0.0688 lgN	0.99	1.89
R40S0.5	0.5	lg*S* = 0.16223–0.06995 lgN	1.00	2.15
R40S1.5	0.5	lg*S* = 0.15254–0.06835 lgN	1.00	2.34
NAC	0.7	lg*S* = 0.11265–0.06014 lgN	0.97	2.40
R20	0.7	lg*S* = 0.12902–0.06399 lgN	0.98	2.16
R20S0.5	0.7	lg*S* = 0.15653–0.0688 lgN	1.00	2.36
R20S1.5	0.7	lg*S* = 0.10494–0.05797 lgN	0.99	2.78
R40	0.7	lg*S* = 0.12864–0.06656 lgN	0.99	1.85
R40S0.5	0.7	lg*S* = 0.13803–0.06647 lgN	1.00	2.14
R40S1.5	0.7	lg*S* = 0.11976–0.06365 lgN	1.00	2.32
NAC	0.9	lg*S* = 0.08534–0.05627 lgN	0.95	2.38
R20	0.9	lg*S* = 0.13715–0.06818 lgN	1.00	2.07
R20S0.5	0.9	lg*S* = 0.14913–0.06991 lgN	1.00	2.28
R20S1.5	0.9	lg*S* = 0.09425–0.05781 lgN	1.00	2.72
R40	0.9	lg*S* = 0.0924–0.0615 lgN	0.98	1.83
R40S0.5	0.9	lg*S* = 0.12321–0.06533 lgN	1.00	2.10
R40S1.5	0.9	lg*S* = 0.07639–0.05702 lgN	0.99	2.31

**Table 8:** Fiber parameters under different survival probabilities.

P	Fatigue Equation	R^2^
0.5	*S* = 1.173 + 0.172$\lambda_{f}\;$ − 1.037$\lambda_{f}^{2}$ − 0.103 lgN	0.984
0.7	*S* = 1.146 + 0.242$\lambda_{f}$ − 1.525$\lambda_{f}^{2}$ − 0.101 lgN	0.982
0.9	*S* = 1.11 + 0.313$\lambda_{f}$ − 2.108$\lambda_{f}^{2}$ − 0.097 lgN	0.971

**Table 9:** Fatigue strain equation parameters.

ε	NO.	S	*e*	*f*	*g*	R^2^
ε_max_	R20	0.6	9.77211 × 10^−5^	0.068	−0.18992	0.9752
		0.7	1.20 × 10^−4^	0.07315	−0.18941	0.99518
		0.8	1.26 × 10^−4^	0.08629	−0.20758	0.99597
	R20S0.5	0.6	8.41 × 10^−5^	0.02535	−0.26136	0.96377
		0.7	1.49 × 10^−4^	0.16966	−0.15546	0.99883
		0.8	1.56 × 10^−4^	0.13298	−0.18807	0.9989
	R20S1.5	0.6	1.25 × 10^−4^	0.11264	−0.20144	0.989
		0.7	1.65 × 10^−4^	0.14207	−0.13586	0.997
		0.8	1.63 × 10^−4^	0.0938	−0.19764	0.987
	R40	0.6	9.23 × 10^−5^	0.10095	−0.1709	0.988
		0.7	1.14 × 10^−4^	0.10224	−0.15774	0.992
		0.8	1.23 × 10^−4^	0.11624	−0.18064	0.985
ε_r_	R20	0.6	2.33 × 10^−5^	0.16396	−0.28888	0.99339
		0.7	5.64 × 10^−5^	0.26793	−0.24083	0.986
		0.8	7.44 × 10^−5^	0.23824	−0.20247	0.995
	R20S0.5	0.6	2.90 × 10^−5^	0.15181	−0.2653	0.997
		0.7	4.76 × 10^−5^	0.11983	−0.22689	0.9567
		0.8	7.50 × 10^−5^	0.21628	−0.16616	0.997
	R20S1.5	0.6	3.67 × 10^−5^	0.12126	−0.26958	0.99566
		0.7	6.60 × 10^−5^	0.1482	−0.18465	0.9755
		0.8	8.30 × 10^−5^	0.18668	−0.20283	0.99309
	R40	0.6	1.53 × 10^−5^	0.09551	−0.41742	0.98213
		0.7	5.76 × 10^−5^	0.19868	−0.289	0.989
		0.8	6.86 × 10^−5^	0.19053	−0.25309	0.99814

**Table 10:** Damage variable fitting parameters.

NO.	S	*e*′	*f*′	*g*′	R^2^
R20	0.6	0.24058	0.51221	−0.31232	0.98
	0.7	0.31507	0.60662	−0.25607	0.99
	0.8	0.38653	0.5237	−0.22125	0.99
R20S0.5	0.6	0.20716	0.52728	−0.35371	0.981
	0.7	0.30548	0.51301	−0.29834	0.977
	0.8	0.38439	0.55781	−0.16991	0.96142
R20S1.5	0.6	0.19946	0.46381	−0.34634	0.996
	0.7	0.29451	0.46071	−0.22484	0.993
	0.8	0.37852	0.51185	−0.19003	0.982
R40	0.6	0.26891	0.45785	−0.45067	0.99
	0.7	0.32663	0.62554	−0.27886	0.982
	0.8	0.40222	0.56091	−0.24417	0.998

## Data Availability

The original contributions presented in this study are included in the article. Further inquiries can be directed to the corresponding author.
